# Early sofosbuvir-ledipasvir treatment for acute HCV infection induced severe immune thrombocytopenia – a case report

**DOI:** 10.1186/s12879-018-3597-4

**Published:** 2018-12-19

**Authors:** Vincent Alcazer, Patrick Miailhes, Christophe Ramière, Caroline Charre, Laurent Cotte

**Affiliations:** 1 Département d’hématologie clinique, Hospices Civils de Lyon, Centre Hospitalier Lyon Sud, 69495 Pierre-Bénite, France; 2 Inserm U1052, Centre de Recherche en Cancérologie de Lyon, 69008 Lyon, France; 3 Service des maladies infectieuses et tropicales, Hospices Civils de Lyon, Hôpital de la Croix Rousse, 69004 Lyon, France; 4 Laboratoire de virologie, Hospices civils de Lyon, Hôpital de la Croix Rousse, 69004 Lyon, France; 5 INSERM U1111, Centre International de Recherche en Infectiologie, Lyon, France

**Keywords:** Hepatitis C virus, Sofosbuvir-ledipasvir, Acute immune thrombocytopenia

## Abstract

**Background:**

Hepatitis C virus (HCV) infection is a recognised cause of secondary immune thrombocytopenia (ITP). While its incidence has been largely described during chronic HCV infection, only one case of ITP secondary to acute HCV infection has been reported at this time.

**Case presentation:**

We report herein the case of severe ITP secondary to an acute HCV genotype 1a reinfection in a human immunodeficiency virus (HIV)-negative man having sex with men who had been cured several years before of a previous acute genotype 4d HCV infection. After an unsuccessful standard therapy with two courses of intravenous immunoglobulin (at 1 g/kg daily for 2 days) associated with methylprednisolone 1 mg/kg daily, antiviral treatment with sofosbuvir-ledipasvir rapidly achieved virological response and normalised the platelet count.

**Conclusions:**

As a direct effect of HCV on megakaryocytes could be the predominant cause of ITP during acute infection, early antiviral treatment may be beneficial in this case.

**Electronic supplementary material:**

The online version of this article (10.1186/s12879-018-3597-4) contains supplementary material, which is available to authorized users.

## Background

Immune thrombocytopenia (ITP) is an acquired thrombocytopenia caused by immune platelet destruction. Most adults (80%) present a primary ITP in which different mechanisms contribute to a reduced platelet lifespan, involving mostly antibody- or T cell-mediated platelet destruction and impaired megakaryocytopoiesis [1]. Secondary ITP pathogenesis is slightly different and relates to underlying disorders among which infections, auto-immune diseases, and lymphoproliferative disorders are the leading causes [2].

Hepatitis C virus (HCV) infection is a recognised cause of secondary ITP [3]. Its occurrence has been well described in the case of chronic infection where the diagnosis can be difficult as different factors can contribute to thrombocytopenia including cross-reactive antibodies directed against platelet antigens, bone-marrow viral infection of progenitor cells, decreased production of thrombopoietin and splenic sequestration secondary to portal hypertension [2]. However, few cases of ITP secondary to acute hepatitis C virus (HCV) infection have been reported to date.

Herein we report the case of a patient with severe secondary ITP during an acute HCV genotype 1a reinfection. Early treatment with sofosbuvir-ledipasvir allowed the prompt recovery of thrombocytopenia.

## Case presentation

In July 2017, a 54-year-old male was hospitalised for the recent appearance of multiple purpuric spots on the legs associated with gum bleeding. The patient was an HIV-negative man having sex with men, receiving HIV pre-exposure prophylaxis with tenofovir/emtricitabine for over a year. His medical history was significant for a primary syphilis in 2014 and multiple episodes of urethritis in the recent years. He reported frequent unprotected anal sex, with occasional bleeding, insertive and receiving fisting without gloves, and the use of nasal mephedrone during sexual encounters. He was previously diagnosed with an acute genotype 4 HCV infection in 2011 (Versant HCV genotype 2.0 assay (LiPA), Siemens Healthineers, Erlangen, Germany), cured following a 6-month course of pegylated interferon (IFN) and ribavirin. Acute genotype 1a HCV reinfection was diagnosed on 2017, June 26th (NS5A Sanger sequencing), while HCV-RNA was still negative on 2017, April 4th (Abbott RealTime HCV, Abbott, Molecular, Des Plaines, USA). The patient was asymptomatic at that time, platelet count was normal and HCV-RNA surveillance was scheduled, following the recommendations from the European AIDS clinical society [4].

**Fig. 1:**
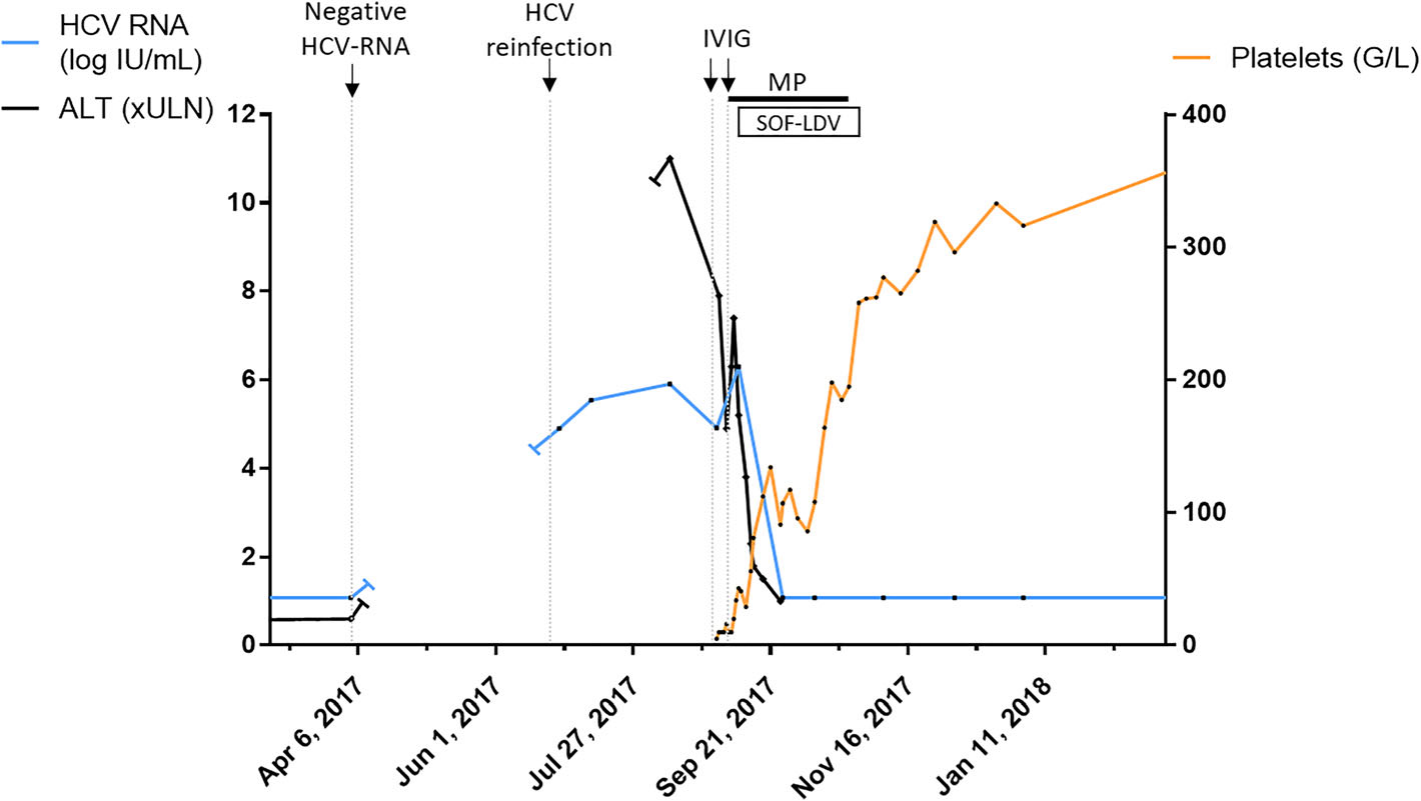
Platelet and HCV viral load. Acute genotype 1a HCV reinfection was diagnosed on August 2017 on a systematic survey, with 11 xULN ALT and 5.91 log IU/mL HCV RNA. Platelets were found at 5 G/L for the unit admission on August 30, 2017. First course of IVIG was administrated on August 31, at the dose of 1 g/kg on day 1 and day 3, with clinical benefit but no effect on the platelet count. Second IVIG course was administrated on September 05 in association with methylprednisolone 1 mg/kg daily. Sofosbuvir-ledipasvir was started on September 11, allowing a sustained recovery of the platelet count with a quick viral load control (basal blue line represents an undetectable HCV viral load, inferior to 12 IU/mL). IVIG: intravenous immunoglobulin, MP: methylprednisolone, SOF-LDV: sofusbuvir-ledispavir, ALT: Alanine transaminase, HCV: Hepatitis C virus

Initial physical examination found no other symptoms apart from a petechial purpura of the lower extremities and oral haemorrhagic blister. Blood pressure was 130/97 mmHg, heart rate 60 bpm with no fever, lymphadenopathy, or splenomegaly. There was no evidence of severe haemorrhage. Laboratory data at the admission are resumed in Table 1. Complete blood count found a severe thrombocytopenia (5 G/L) without any other cytopenia. Thrombocytopenia was confirmed on the peripheral blood smear which exhibited no morphological abnormalities and the absence of schistocytes. No other associated haemostasis abnormality was present (normal fibrinogen and factor V). Serum protein electrophoresis found no clonal gammopathy. Thyroid-stimulating hormone was normal. HIV serology was repeatedly negative and Hepatits B surface antibodies were detectable following a previous vaccination. At that time, mild elevation in aspartate transaminase (AST, 6 times the upper limit of normal (ULN)) and alanine transaminase (ALT, 7 x ULN) was found associated with a detectable HCV viral load of 4.9 log IU/mL (Fig. 1). There was no evidence of other replicating viruses including cytomegalovirus, parvovirus B19, human herpes virus type 6 and type 8 (except a slight Epstein-Barr virus replication which was considered insignificant (348 UI/mL). Cryoglobulin investigation was negative. No circulating anti-platelet IgG antibodies were detected. A bone marrow aspiration was performed; normal cellularity with an increased number of megakaryocytes and normal erythropoiesis and myelopoiesis was found, thus confirming the diagnosis of ITP secondary to acute HCV infection.

**Table 1:** Laboratory data at the admission

Parameter	Baseline value	Normal value (range)
Complete blood count		
Leucocytes	5.69 G/L	4.00–10.00
Haemoglobin	149 g/L	130–170
MCV	95.0 fL	80–100
Platelet	< 10 G/L	150–400
Schistocytes	< 1%	< 1%
Neutrophils	3.27 G/L	1.8–7.5
Eosinophils	0.19 G/L	0.02–0.8
Lymphocytes	1.65 G/L	1–4
Monocytes	0.52 G/L	0.2–0.9
Haemostasis	Normal PT and aPTT	
Fibrinogen	2.23 g/L	2.13–4.22
Factor V	> 150%	61–142
Blood chemistry		
Creatinine	86 μmol/L	59–104
eGFR	87.8 mL/min/1.73 m^2^	90.0–120.0
AST	202 IU/L	15–37
ALT	483 IU/L	16–61
Alkaline phosphatase	101 IU/L	50–136
GGT	292 IU/L	15–85
Total bilirubin	13 μmol/L	3–17
Conjugated bilirubin	4 μmol/L	0–3
Lacticodehydrogenase	254 IU/L	87–241
Serum protein electrophoresis	Normal	
Cryoglobulin	Negative	
Myelogram	Normal cellularity	
Serology		
Syphilis	Negative	
HIV	Negative	
HBV	Positive for Hbs Ab	
CMV	Positive for IgG	
EBV	Positive for IgG	
Blood / plasma viral load		
HCV RNA	82,004 IU/mL	< 12
CMV DNA	0 IU/mL	150–500
EBV DNA	348 IU/mL	182–500
HIV RNA	< 40 copies/mL	< 40
Parvovirus B19 DNA	0 copies/mL	70–87
HHV6 DNA	0 copies/mL	
HHV8 DNA	0 copies/mL	500–2000

*GGT* Gamma Glutamine Transferase, *PT Prothrombine time, aPTT Activated Partial Thromboplastin Time*

An initial administration of intravenous immunoglobulin (IVIG; at the dose of 1 g/kg on day 1 and day 3) had clinical benefit (regression of gum bleeding and purpura), but did not have any effect on the platelet count which remained below 10G/L. A second administration of IVIG (1 g/kg daily for 2 days) combined with methylprednisolone (1 mg/kg daily) improved platelet count which reached 40G/L but a normal value was not attained (Fig. 1). At which point it was decided after collegial discussion to start treating the infectious trigger. Pegylated IFN and ribavirin combination was contra-indicated because of severe thrombocytopenia, therefore a 12 weeks course of sofosbuvir-ledipasvir was indicated. At initiation of the treatment, the platelet count was 43 G/L and HCV viral load was 6.3 log IU/mL. At day 15 of antiviral treatment, HCV viral load was undetectable (< 12 IU/ml) and the platelet count had increased to 108 G/L allowing rapid tapering and discontinuation of corticosteroids before the end of antiviral therapy. HCV viral load remained undetectable until week 12 post-treatment, confirming HCV cure (Fig. 1). Platelet count fully normalised 5 weeks following treatment initiation and remained within normal range thereafter. The patient went through his 12 weeks course of sofosbuvir-ledipasvir with a good tolerance, without any specific adverse event reported.

## Discussion and conclusions

Chronic HCV infection has been associated with immune thrombocytopenia; the incidence is estimated to be 30.2–53 cases / 100,000 person-years [5, 6]. Conversely, HCV antibodies have been found in 10 to 36% of patients with chronic ITP in cross-sectional studies [5, 6]. However, our case is a rare report of ITP in the context of acute hepatitis C. Only one similar case has already been reported by Narita et al. in 2003 [7]. According to this report, ITP also occurred at the second HCV infection after a first HCV infection cured by 6 months of IFN-α2b. HCV genotype was found to be 2a in this reinfection case, versus 2b for the first one. In contrary to our findings, platelet associated immunoglobulins (PAIgG) were found positive in this report, with no other circulating antibodies (antinuclear antibody and rheumatoid factor were negative). Additionally, platelet count increased up to 39,000 /μL spontaneously and the PAIgG returned to the normal ratio without any specific therapy while in our case, platelet count only recovered after specific antiviral therapy. Considering these differences, a distinct physiopathological mechanism may have been the source of ITP in these two cases.

Thrombocytopenia is frequent in viral infection. In such situations, a complex crosstalk between platelet and the virus is engaged and different mechanisms may contribute to two major events [8]: first, a decreased platelet production which can be induced by direct infection of megakaryocytes, infection of haematopoietic stem cells, induction of a type-1 IFN response leading to suppression of platelet formation, or modulation of liver thrombopoietin production; second, an increased platelet destruction, either by a direct contact during viraemia, interaction with immune complexes, cross-reactive antibodies directed against the virus, or proinflammatory events induced by the infection [8].

The exact mechanism of HCV-induced ITP is still not clear. Extra-hepatic manifestations are common in HCV-infected patients, most of which are immune-related manifestations including mixed cryoglobulinaemia, arthralgia/myalgia, and auto-antibody production [9]. Importantly, the presence of only anti-platelet antibodies is not associated with thrombocytopenia [10]. This leads to consider the potential role of immune complexes promoted by the presence of cross-reactive antibodies or a compensating mechanism following a decrease in thrombopoietin production [10]. A direct effect of the virus on megacaryocytes may also be suspected as HCV can bind to CD81 on the platelet membrane, and HCV-RNA has also been detected in washed platelets of infected patients [11]. Platelets could thus either favour the spread of the virus or contribute to its immune recognition by providing targetable antigens [10]. Moreover, detection of HCV-RNA with a higher frequency in platelets of thrombocytopenic patients suggest that HCV is directly involved in this process [12]. At this time, there is no evidence for a particular association between specific HCV genotypes and incidence of ITP [12].

The treatment of HCV-related ITP, particularly in the case of acute infection, is not standardized. Dufour et al. reported the outcome of 8 patients with HCV-related ITP [13]. Patients had a poor response to initial corticosteroids therapy, with only one complete response and three partial response. IVIG led to transient efficacy in three other patients. Of eight patients treated by antiviral therapy associating IFN-α with ribavirin, five responded. Normalisation of platelet count occurred in three responders, and normalisation of viral load was usually slow. Other authors have reported the increase of platelet count associated with complete HCV eradication after IFN therapy [14, 15]. However, kinetic of platelet recovery was usually low in these cases.

Herein, we describe the case of a severe ITP associated with acute HCV reinfection. IVIG was chosen as the first-line therapy, owing to their efficacy in primary ITP and secondary ITP related to chronic HCV infection [6]. The absence of effect on the platelet count led to a second course of IVIG and administration of methylprednisolone, which improved only slightly the thombocytopenia. Sofosbuvir-ledipasvir treatment was then initiated which rapidly decreased viral load to under the limit of detection and normalised the platelet count. Interestingly, no drop back of platelet following corticosteroid discontinuation was observed. The acute installation of the thrombocytopenia, its refractoriness to the first-line immunomodulatory and immunosuppressive therapy, the rapid improvement following antiviral treatment and the absence of circulating anti-platelet antibodies suggest a predominant effect of direct viral-mediated platelet destruction in this context where the other adaptive mechanisms might not had the time to develop. Notably, the first HCV infection in the patient presented herein did not trigger any thrombocytopenia. There is currently no data on the risk on ITP following HCV reinfection and we can only speculate on the relation between the first infection and the ITP case.

In summary, we report herein the case of a secondary ITP associated with an acute genotype 1a HCV reinfection in a 54-year-old patient. Early treatment with sofosbuvir-ledispavir was followed by a rapid virological response and a sustained platelet count recovery, suggesting a viral-mediated platelet destruction corrected by the antiviral therapy.

## Additional file

Additional file 1: **Table S1.** Laboratory data evolution. (XLSX 11 kb)

## Abbreviations

HCV: Hepatitis C Virus
ITP: Immune thrombocytopenia
IVIG: Intravenous Immunoglobulin
